# On Some Practical Points in Treatment of Syphilis

**Published:** 1890-04

**Authors:** 


### TAYLOR (R. W.) ON SOME PRACTICAL POINTS IN THE TREATMENT OF SYPHILIS.

The author is not one of those who commence specific treatment as soon as the character of the initial lesion is made out. According to him, "syphilis is not mature until the date of secondary manifestations, when the newly formed, young, round, infecting cells are proliferated in vast quantities and are thrown into the general circulation, and by it carried throughout the body. when this has occurred, I think syphilis may be said to be "ripe"; then, and not till then, we have something tangible to treat. At this time, mercury introduced into the organism can exert its marvellous powers in destroying this, then, young, nascent, infectious material, and in causing its absorption."

He thinks we are unable to abort the disease in its first stage.

Ignoring the expectant and the spasmodic or interrupted method of treatment, the author advocates the continuous and tonic treatment of the disease. He favors the internal use of the green iodide in doses of from 1-4 to 1-2 a grain three times a day in pill form, or from 1-2 to 1 grain doses of the tannate of mercury, the dosage of either remedy depending on the age and robustness of the patient.

The general health of the patient must be kept at the very highest point, the soundness of the mouth, pharynx, and stomach being an object of especial anxiety.

He favors inunctions at times as a change, and particularly when cutaneous lesions are present, having satisfied himself that the local action of the remedy is salutary.

He remits treatment occasionally when a lull in virulent manifestations occurs, lengthening the intervals in the second year, when he combines his remedy with some iodide of potassium.

He believes that at times there is an advantage in bringing the application near the lesion, hence he frequently uses inunctions over or hypodermatic injections about indolent glandular enlargements, inunctions about the neck and jaws, temple and occiput, in early and late meningeal and cerebral disease, and after cleansing antiseptically mucuous patches and condylomata, he dusts them with calomel.

In some cases where rapid action is necessary, when the lesions are on the face, neck, hand, etc., the author resorts to the subcutaneous employment of the drug, but he is wisely conservative in this use of the remedy, and countenances only the employment of a soluble preparation in this way.—*Med. News*, Dec. 7th, 1889—*Medical Analectic Epitome.*

---

THE LATEST STRATEGY OF A PARIS PAPER for attracting readers is the engagement of two eminent physicians to attend gratuitously upon its annual subscribers. Recently the manager of the paper gave notice to one of the physicians "not to prescribe for B any more; his subscription has expired." The doctor replied: "So also has B."

